# Supplementary material for: California air resources board forest carbon protocol invalidates offsets
Source: PeerJ. 2019 Sep 23;7:e7606. doi: 10.7717/peerj.7606 (PMC6761920; doi:10.7717/peerj.7606)
Supplement: Supplemental Information 1 — The project locations are displayed for the locations of 59 NEE1 and 63CARB-CAR project sites. Information for each site, including the underlying biome and land cover classification. A toggle is provided to move between the screens with biomes, land cover and boundary lines. Base Map: © Mapbox© OpenStreetMap. Data: U.S. EPA Office of Research & Development (ORD) - National Health and Environmental Effects Research Laboratory (NHEERL). Imagery: © DigitalGlobe. [file peerj-07-7606-s001.docx]

**Supplement S1: Project Locations Interactive Map.** The interactive map can be accessed here: <http://geocambridge.com/PEMfluxmap/>

####
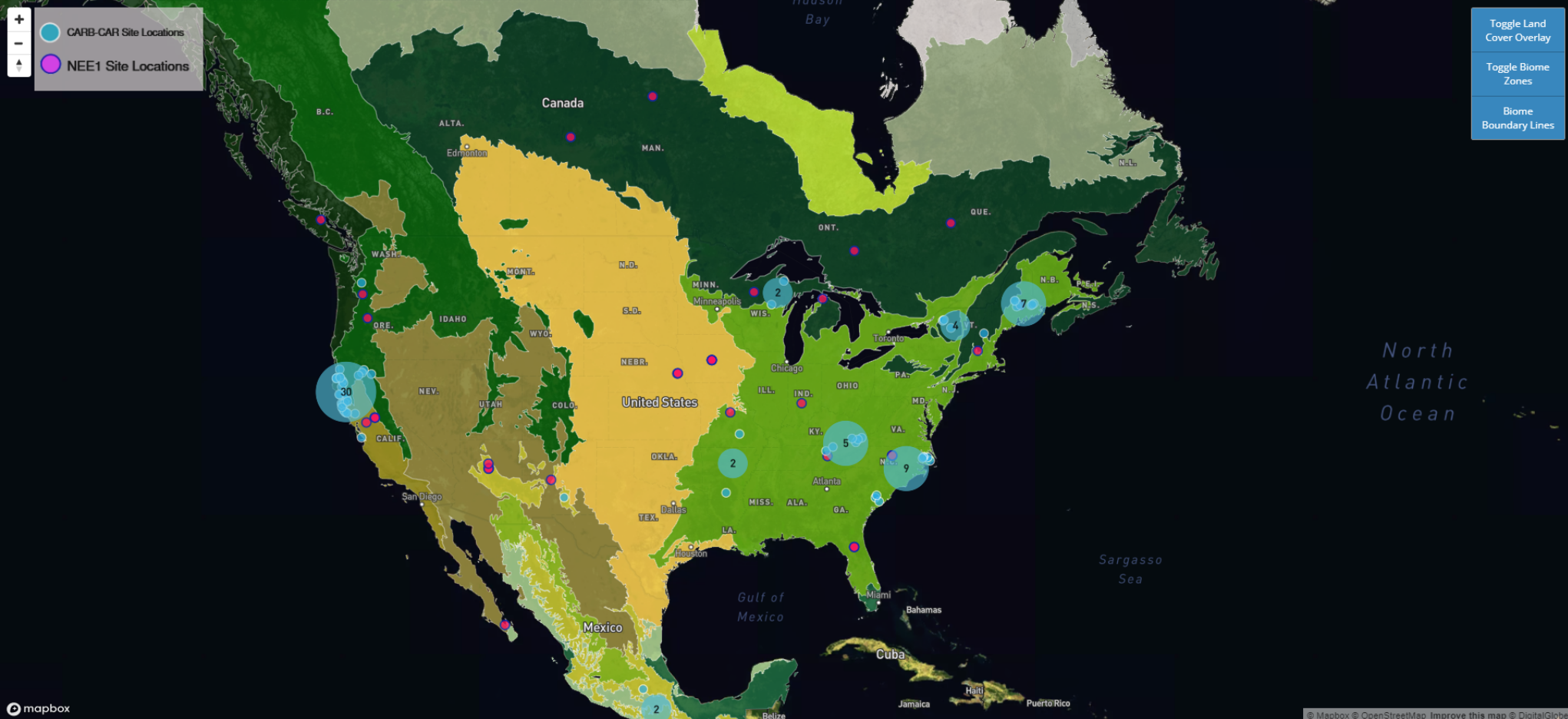


The project locations are displayed for the locations of 59 NEE1 (D. Baldocchi, Chu, and Reichstein 2018) and 63 CARB-CAR project sites (see Methods section for details). Information for each site, including the underlying biome and land cover classification is provided, allowing users to compare the underlying ecological properties for a given project site with other sites. A toggle is provided to move between the screens with biomes, land cover and boundary lines. Each project site point is geocoded or otherwise plotted in WGS84 coordinate system using the best available latitude and longitude information for each location. Blue circles represent NEE1, pink circles represent CARB-CAR projects. All sites on the map feature a unique site identifier code (e.g., CAR697)  which is linked to project data in Table 1 and summarized in Table I, Supplements 3-5. Clusters of sites are shown in blue circles. A link to the project web page for each site is included in the pop-up display. CARB-CAR and NEE1 sites were classified for functional type based on the 2015 Climate Change Initiative Land Cover (CCI-LC) dataset ([https://www.esa-landcover-cci.org](https://www.esa-landcover-cci.org/)). This Land Cover product was developed by the European Space Agency Climate Change Initiative (ESA CCI Land Cover project). The land cover overlay represents a 300-meter resolution global land cover classification and is compatible with the plant functional types used in many global land cover models. The base map provided is Satellite Streets by Mapbox ([https://www.mapbox.com](https://www.mapbox.com/)). Primary biome classification data is displayed using Level I Ecoregions for North America, published by the US EPA Commission for Environmental Cooperation. This classification represents the highest level of ecologically distinct areas and is divided among 15 classifications. This provides a broad level classification for the underlying ecosystem, grouping similar discrete biomes and establishing boundaries between transitional zones. The PEM FluxMap provides access to both Level I Ecoregion data, and satellite based land cover classification data (at a much finer spatial resolution), along with satellite and orthophoto based imagery, to demonstrate a combination of the best available information for identifying the underlying environmental resources, in an interactive, spatial format. Interactive map attributions: 1) The Geocambridge and PEMfluxmap URL, image displayed above, interactive functionality and codes employed to produce the above are research products of PEM. 2) Mapbox is open source software providing the basemap (<https://www.mapbox.com/about/open/>), attribution is labeled within the image screen. 3) The GIS land cover overlay shown is provided by the US EPA, open source metadata (<https://www.data.gov/open-gov/>) and is available here:

<ftp://newftp.epa.gov/EPADataCommons/ORD/Ecoregions/cec_na/NA_CEC_Eco_Level1.htm>.
