# Supplementary material for: California air resources board forest carbon protocol invalidates offsets
Source: PeerJ. 2019 Sep 23;7:e7606. doi: 10.7717/peerj.7606 (PMC6761920; doi:10.7717/peerj.7606)
Supplement: Supplemental Information 2 — Statistical methods are described for Figs. 1–3 and Table 2. [file peerj-07-7606-s002.docx]

#### **Supplement S2: Statistical Analyses**

Figure 1. The CARB-CAR dataset consists of 340 sample points spanning the years 2001-2014. The NEE1 dataset consists of 540 sample points spanning over the years 1992-2015. The skewness and kurtosis of the CARB-CAR and NEE1 datasets are -3.69 and 17.67, respectively. As a comparison, the skewness and kurtosis for the NEE1 dataset are 0.25 and 2.31.

The skewness is calculated in the following way:

$$\frac{n\sum_{i=1}^{n} {(x_{i}-\overline{x})}^{3}}{(n-1)(n-2)s^{3}}$$

where $\overline{x}$ and s are the sample mean and sample standard deviation of the CARB-CAR data, and n=340. The skewness is negative, which means that the distribution is skewed to the left. The kurtosis is calculated in the following way:

$$\frac{n(n+1)\sum_{i=1}^{n} {(x_{i}-\overline{x})}^{4}}{(n-1)(n-2)(n-3)s^{4}}-\frac{3{(n-1)}^{2}}{(n-2)(n-3)}$$

where $\overline{x}$ and s are the sample mean and sample standard deviation of the CARB-CAR data, and n=340. It provides a measurement of the extremities of the data. A kurtosis value of 17.67 demonstrates the presence of very large outliers.

Figure 2. We calculate the 95% confidence interval for the difference in means of the two data sets CARB-CAR and NEE1. The first bar is based on the complete data sets over all available years. We use the following formula for large sample size:

$$\left( \overline{x_{1}}-\overline{x_{2}} \right)\pm1.96\sqrt{\frac{s_{1}^{2}}{n_{1}}+\frac{s_{2}^{2}}{n_{2}}}$$

where $\overline{x_{1}}$ and $\overline{x_{2}}$ are the sample means, and $s_{1}$ and $s_{2}$ are the sample standard deviations of the two samples.

For the year 2007, we have 23 CARB-CAR and 42 NEE1 data points. For the year 2008, we have 24 CARB-CAR and 41 NEE1 data points. In order to calculate the confidence interval, we use the following formula for a small sample size:

$$(\overline{x}_{1}-\overline{x}_{2})\pm t\sqrt{s_{p}^{2}\left( \frac{1}{n_{1}}+\frac{1}{n_{2}} \right)}$$

where

$$s_{p}^{2}=\frac{\left( n_{1}-1 \right)s_{1}^{2}+(n_{2}-1)s_{2}^{2}}{n_{1}+n_{2}-2}$$

and t is based on $(n_{1}+n_{2}-2)$ degrees of freedom.

Table II. Table II shows the results of multiple one-sided hypothesis tests, ranging from 2002 to 2014. For each year, we test the following hypotheses:

$$H_{0}: \mu_{1}-\mu_{2}\leq D$$

$$H_{a}: \mu_{1}- \mu_{2}>D$$

where $\mu_{1}$ and $\mu_{2}$ are the true population means and D is the allowed 5% threshold. Since the CARB-CAR sample sizes vary from 2 to 32 per year, we use a small-sample one-sided hypothesis test. The test statistic is the following:

$${t=\frac{\left( \overline{x}_{1}-\overline{x}_{2} \right)-D}{\sqrt{s_{p}^{2}}\sqrt{\left( \frac{1}{n_{1}}+\frac{1}{n_{2}} \right)}}}$$

where $s_{p}^{2}$ was already defined in the description of figure 3 methodology, and t is based on $(n_{1}+n_{2}-2)$ degrees of freedom.
