# Supplementary material for: California air resources board forest carbon protocol invalidates offsets
Source: PeerJ. 2019 Sep 23;7:e7606. doi: 10.7717/peerj.7606 (PMC6761920; doi:10.7717/peerj.7606)
Supplement: Supplemental Information 8 — The equations for net GHG carbon balance as defined by the CARB-CAR protocols (California Air Resources Board, 2014; California Air Resources Board, 2015a; California Air Resources Board, 2015b) are analyzed considering the simple case of a one-year project. Related forest protocol equations (ACR, CDM, VCS) are also summarized and reviewed. We show that protocol equations exclude efflux of CO2 soil carbon and ecosystem respiration (e.g., Reco) in the CARB-CAR and related forest carbon protocols. [file peerj-07-7606-s008.docx]

**Supplement S6. The CARB-CAR GHG Net Quantification Equations**

The equations for material carbon balance as defined by the CARB-CAR protocols (California Air Resources Board, 2014, 2015) are analyzed considering the simple case for a one-year project. Related forest protocol equations (ACR, CDM, VCS) are also summarized and reviewed. We show that equations exclude efflux of CO_2_ soil carbon and ecosystem respiration (e.g., R_eco_) in the CARB-CAR and related forest carbon protocols. The terms employed by the CARB-CAR and related protocols cannot be clearly defined as GPP or NEE in the absence of a term for ecosystem respiration (Supplement S7). The net GHG reductions and GHG removal enhancements are provided by the common net quantification equation provided by CARB-CAR (California Air Resources Board, 2011, p. 37, Equation 6.1; California Air Resources Board, 2014 p. 39, Equation 6.1; (California Air Resources Board, 2015b, p. 47, Equation 5.1; (Climate Action Reserve, 2018, p. 46, Equation 6.1).

Net GHG Reductions and GHG Removal Enhancements

$\boldsymbol{QR}_{\boldsymbol{y}}\boldsymbol{=}\left[ \left( \boldsymbol{\Delta}\boldsymbol{C}_{\boldsymbol{onsite}}\boldsymbol{-}\boldsymbol{\Delta}\boldsymbol{B}\boldsymbol{C}_{\boldsymbol{onsite}} \right)\boldsymbol{+}\left( \boldsymbol{A}\boldsymbol{C}_{\boldsymbol{wp, y}}\boldsymbol{- B}\boldsymbol{C}_{\boldsymbol{wp,y}} \right)\boldsymbol{\times80\%+S}\boldsymbol{E}_{\boldsymbol{y}} \right]\boldsymbol{\times}\left( \boldsymbol{1-ACD} \right)\boldsymbol{+}\boldsymbol{N}_{\boldsymbol{y-1}}$ (1)

Equation *(1)* can be rewritten for clarity with each grouped expression or factor given a subscript for discussion purposes written as (for the definition of each term above see(California Air Resources Board, 2015)),

${QR}_{y}= [(actual onsite carbon-baseline onsite carbon)_{1}+(actual onsite wood products-baseline onsite wood products)_{2}\times(80\%)_{3}+(secondary emissions)_{4}]\times(avoided conversion factor)_{5}+ (negative carry over)_{6}$ (2)

Expression *(2)* can be simplified considering the case of a single year as,

$\boldsymbol{QR}_{\boldsymbol{y}}\boldsymbol{=}\left[ \left( \boldsymbol{\Delta}\boldsymbol{C}_{\boldsymbol{onsite}}\boldsymbol{-}\boldsymbol{\Delta}\boldsymbol{B}\boldsymbol{C}_{\boldsymbol{onsite}} \right)_{\boldsymbol{1}}\boldsymbol{+}\left( \boldsymbol{0}_{\boldsymbol{wp, y}}\boldsymbol{-}\boldsymbol{0}_{\boldsymbol{wp,y}} \right)_{\boldsymbol{2}}\boldsymbol{\times}\boldsymbol{0\%}_{\boldsymbol{3}}\boldsymbol{+}{\boldsymbol{0}_{\boldsymbol{y}}}_{\boldsymbol{4}} \right]\boldsymbol{\times}\left( \boldsymbol{1-0} \right)_{\boldsymbol{5}}\boldsymbol{+}\boldsymbol{0}_{\boldsymbol{6}}\boldsymbol{,}$ (3)

and by considering term 2, “wood products”, as zero product in this case. Zero wood product eliminates terms 3 and 4. Term 5 is set at zero reflecting no project conversion in this case. Expression *6*, carryover of GHG reductions from a previous year, is set at zero considering this year as a one-year project for the purposes of illustration. The simplifications noted can be expressed as,

$\boldsymbol{QR}_{\boldsymbol{y}}\boldsymbol{=}\left( \boldsymbol{actual onsite carbon-actual baseline carbon} \right).$ (4)

The terms for actual onsite carbon and actual baseline carbon are defined as follows:

$AC_{onsite, y}$= Actual onsite carbon (CO_2_e) as inventoried for year $y$, and, (5)

$BC_{onsite,y}$ = Baseline onsite carbon (CO_2_e) as estimated for year *y.*  (6)

Both “actual onsite” and “baseline onsite” carbon terms require an inventory of required carbon pools as identified in tables for reforestation (Table 4.1(California Air Resources Board, 2015)), improved forest management (Table 4.2(California Air Resources Board, 2015)) and for avoided conversion (Table 4.3(California Air Resources Board, 2015)) and counterfactual arguments to establish baselines. The carbon inventory tables identify the following carbon pools: 1) standing live tree carbon, 2) shrubs and herbaceous understory carbon, 3) standing dead tree carbon, 4) lying dead tree carbon, 5) litter and duff carbon, 6) soil carbon, 7) carbon in in-use forest products, 8) forest product carbon in landfills, 9) biological emissions from site preparation activities, 10) mobile combustion emissions from site preparation activities, 11) mobile combustion activities from ongoing project operation and maintenance, 12) stationary combustion emissions from ongoing project operation and maintenance, and, 13) biological emissions from clearing of forestland outside of the project area. Each of the above pools is labeled as RF for reforestation, IFM for improved forest management and AC for avoided conversion within each table. Specifically, the soil carbon pool for each project type is labeled as RF-6, IFM-6 and AC-6. In the case for each project type, the pools for RF-6, IFM-6 and AC-6 are noted as “included/excluded” according to project activities such as deep ripping or furrowing and mechanical site preparation not conducted on contours(California Air Resources Board, 2015). Additionally, no crediting is allowed for increased soil carbon(California Air Resources Board, 2015). Thus, in cases where soil carbon is excluded, such as for the projects analyzed in this report and listed in Table I, we can rewrite (4) for clarity as,

$\boldsymbol{QR}_{\boldsymbol{y}}\boldsymbol{=}\left( \boldsymbol{actual onsite carbon}_{\boldsymbol{excluding soil carbon}}\boldsymbol{-}\boldsymbol{actual baseline carbon}_{\boldsymbol{excluding soil carbon}} \right)\boldsymbol{.}$ (7)

Equation (7) can be rewritten as,

$\boldsymbol{QR}_{\boldsymbol{y}}\boldsymbol{=}\left( \boldsymbol{actual onsite above ground carbon}\boldsymbol{-}\boldsymbol{actual above ground baseline carbon} \right)\boldsymbol{.}$ (8)

In summary, the CARB equations, in practice as reported here, exclude terms for soil carbon in carbon pool accounting. Identical equations are employed for the CAR projects(Climate Action Reserve, 2017).

Next, we consider similarities between the CARB-CAR equations and the ACR, CDM and VCS protocols. The American Carbon Registry identifies an analogous equation for net anthropogenic GHG removals by equation 44 of the ACR methodology(*Methodology for the quantification, monitoring, reporting and verification of greenhouse gas emissions reductions and removals from afforestation and reforestation of degraded land about reforestation of degraded land*, 2017):

$\boldsymbol{C}_{\boldsymbol{AR-AC}}\boldsymbol{= \Delta}\boldsymbol{C}_{\boldsymbol{Actual}}\boldsymbol{- \Delta}\boldsymbol{C}_{\boldsymbol{BSL}}\boldsymbol{-LK},$ (9)

Where:

$C_{AR-AC}$ = Net anthropogenic GHG removals by sinks; MT CO_2_e, (10)

$\Delta C_{Actual}$ = Actual net GHG removals by sinks; MT CO_2_e, (11)

$\Delta C_{BSL}$ = Baseline net GHG removals by sinks; MT CO_2_e, and, (12)

$LK$ = Total GHG emissions dues to leakage: MT CO_2_e. (13)

No terms for direct measurement of CO_2_ are identified. Soil carbon is identified as an optional carbon pool.

The Clean Development Mechanism, AR-ACM0003 A/R, for large-scale consolidated methodology for afforestation and reforestation of lands except wetlands, Version 02.0 Sectoral scope(s)(UNFCCC, 2013): 14, states:

$\Delta C_{AR-CDN,t}= \Delta C_{ACTUAL,t}- \Delta C_{BSL,t}-{LK}_{t}$, (14)

Where,

$C_{AR-CDM,t}$ = Net anthropogenic GHG removals by sinks, in year t; t CO_2_-e, (15)

$\Delta C_{ACTUAL,t}$ = Actual net GHG removals by sinks, in year t; t CO_2_-e, (16)

$\Delta C_{BSL,t}$ = Baseline net GHG removals by sinks, in year t, t CO_2_-e, and, (17)

${LK}_{t}$ = GHG emissions due to leakage, in year t, t CO_2_-e. (18)

No terms for direct measurement of CO_2_ are identified. Soil carbon is identified as an optional carbon pool.

The Verified Carbon Standard describes the Methodology for Carbon Accounting for Mosaic and Landscape-scale REDD Projects, VM0006(VERRA, 2015), for quantifying emission reductions and/or removals from activities to reduce unplanned deforestation and forest degradation of the mosaic configuration. The methodology is chosen for comparison to the CAR, ACR and CDM protocols as it can be combined with Improved Forest Management (IFM) and Afforestation, Reforestation and Revegetation (ARR) methodologies to implement a landscape scale Reduced Emissions from Deforestation and Forest Degradation (REDD+) projects. The net anthropogenic GHG removal is summarized as follows:

$\boldsymbol{C}_{\boldsymbol{ANR}}\left( \boldsymbol{t} \right)\boldsymbol{=}\boldsymbol{\Delta C}_{\boldsymbol{ANR}}\left( \boldsymbol{t} \right)\boldsymbol{-}\boldsymbol{\Delta}\boldsymbol{C}_{\boldsymbol{ANR,BSL}} \left( \boldsymbol{t} \right)\boldsymbol{,}$ **(19)**

Where:

$C_{ANR}\left( t \right)$ = Net anthropogenic greenhouse gas removals due to biomass increase in assisted natural regeneration during year 𝑡 [tCO_2_e], (20)

${\Delta C}_{ANR}\left( t \right)$ = Annual change in carbon stocks in all selected carbon pools due to ANR during year 𝑡 [tCO_2_e], (21)

$\Delta C_{ANR,BSL} \left( t \right)$ = Baseline GHG gas emissions or sources during year 𝑡 [tCO_2_e]. (22)

No terms for direct measurement of CO_2_ are identified. Soil carbon is identified as an optional carbon pool.

In each case for the ACR, CDM and VCS, similar above ground and baseline terms are employed reflecting the simplified equation (7) noted above for the CARB-CAR protocols. No terms for direct measurement of CO_2_, soil carbon or soil CO_2_ efflux are employed in the protocol equations cited.

**References**

California Air Resources Board (2011) *Compliance Offset Protocol U.S. Forest Projects*. Available at: https://www.arb.ca.gov/regact/2010/capandtrade10/copusforest.pdf.

California Air Resources Board (2014) *Compliance Offset Protocol U.S. Forest Projects*. Available at: https://www.arb.ca.gov/regact/2014/capandtrade14/ctusforestprojectsprotocol.pdf.

California Air Resources Board (2015) *Compliance Offset Protocol US Forest Projects*. Available at: https://www.arb.ca.gov/cc/capandtrade/protocols/usforest/forestprotocol2015.pdf.

Climate Action Reserve (2017) *Comparison of California ARB Compliance Offset Protocol to Climate Action Reserve Voluntary Offset Project Protocol Version 3.2*. Available at: http://www.climateactionreserve.org/how/california-compliance-projects/compliance-offset-program-documents/.

*Methodology for the quantification, monitoring, reporting and verification of greenhouse gas emissions reductions and removals from afforestation and reforestation of degraded land about reforestation of degraded land* (2017). Available at: https://americancarbonregistry.org/carbon-accounting/standards-methodologies/afforestation-and-reforestation-of-degraded-lands/acr-ar-of-degraded-land-v1-2-2017.pdf (Accessed: 20 May 2018).

UNFCCC (2013) *A/R Large-scale Consolidated Methodology Afforestation and reforestation of lands except wetlands Version 2.0*. Available at: https://cdm.unfccc.int/filestorage/T/H/N/THNRJC15IW4K89UBE6DFZYX23OVP0Q/EB75_repan30_AR-ACM0003_ver02.0.pdf?t=Vkh8bzB2d2xpfDB9RaY6Q_kLoASw2EwFcHDb.

VERRA (2015) *VM0007 REDD+ Methodology Framework (REDD-MF), v1.5*. Available at: http://verra.org/methodology/vm0007-redd-methodology-framework-redd-mf-v1-5/.
