# Supplementary material for: California air resources board forest carbon protocol invalidates offsets
Source: PeerJ. 2019 Sep 23;7:e7606. doi: 10.7717/peerj.7606 (PMC6761920; doi:10.7717/peerj.7606)
Supplement: Supplemental Information 9 — A plot of the annual sums measured in the field for gross primary productivity (GPP) versus ecosystem respiration (Reco) is shown based on NEE1 data (Baldocchi, Chu & Reichstein, 2018) employed for the statistical analyses reported in this study. The mean and standard deviation of the NEE 1 data are shown on the right axis. The plot represents data for 59 locations with a total of 544 site-years for direct eddy covariance measurement of net carbon fluxes in diverse natural and managed ecosystems. The mean and standard deviation for NEE1 is −198.0 ± 261.6 gC m−2yr−1 (r2 = 0.907). [file peerj-07-7606-s009.docx]

**Supplement S7. Plot of Gross Primary Productivity, Ecosystem Respiration and Net Ecosystem Exchange**

A plot of the annual sums measured in the field for gross primary productivity (GPP) versus ecosystem respiration (R_eco_) is shown (open circles and error bars) based on NEE1 data (Baldocchi, Chu, & Reichstein, 2018) employed for the statistical analyses reported in this study. The mean and standard deviation of the NEE 1 data are shown on the right axis. The plot represents data for 59 locations with a total of 544 site-years for direct eddy covariance measurement of net carbon fluxes in diverse natural and managed ecosystems. The reported mean and standard deviation for NEE1 is ‒198.0 ± 261.6 gC m^-2^yr^-1^ (r^2^= 0.907).

NEE is plotted on the right axis as the absolute value of NEE (open rectangle and error bars) to emphasize the relative magnitude of terms for the equation: NEE = GPP + R_eco_. Values for NEE, GPP or R_eco_ that fall outside of the ranges presented are likely not reflective of natural or managed forests. The black and gray rectangle symbols represent the Howland Forest, Maine, USA and the Wind River, Washington State, USA, site NEE1 data, respectively, emphasizing overlapping GPP and R_eco_ functional ranges for forests across the USA.

An updated analysis of eddy covariance field data, NEE2 (Baldocchi & Penuelas, 2019), for 155 sites representing 1,163 site years, reports NEE of ‒156 ± 284 gC m^-2^yr^-1^ and similar ranges for GPP and R_eco_ (r^2^ = 0.839). NEE2 is inclusive of NEE1 data.

For comparison, the CARB-CAR population is characterized by a mean and standard deviation of ‒948.8 ± 1,504.8 gC m^-2^yr^-1^ for annual net forest carbon sequestration.


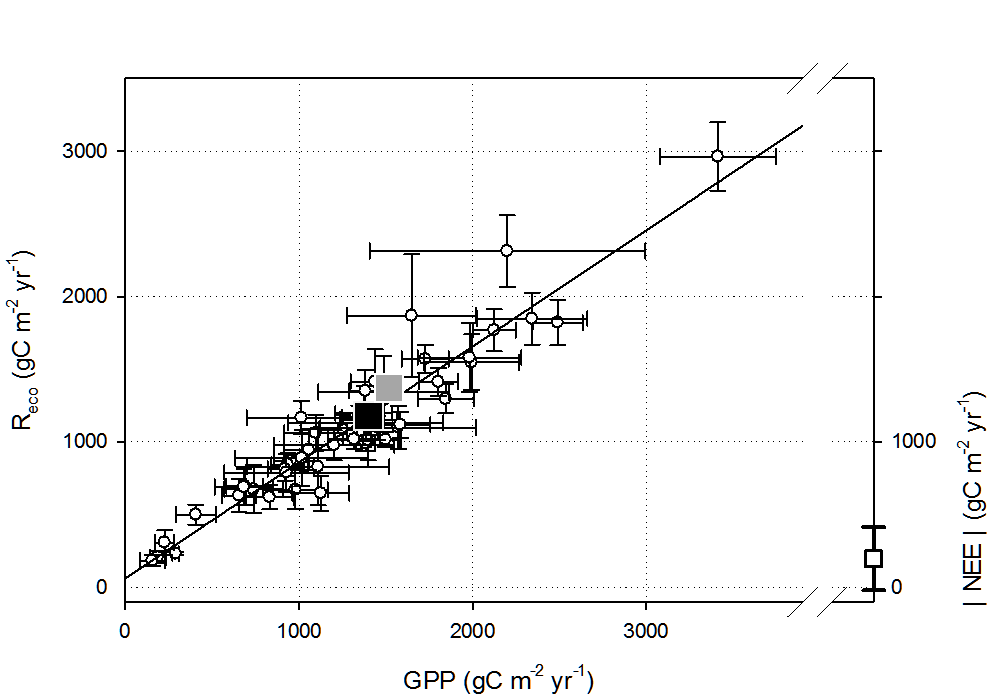


Plot of GPP vs Reco NEE1, open circles (Baldocchi, Chu, & Reichstein, 2018). Absolute value of mean and SD for NEE1 are shown on the right axis, open rectangle. The black and gray rectangle symbols represent the Howland Forest, Maine, USA (Ho-1), and the Wind River, Washington State, USA (Wrc), site NEE1 data, respectively (symbols are enlarged to aid in visualization, data are available in Supplement S4).
