# Supplementary material for: California air resources board forest carbon protocol invalidates offsets
Source: PeerJ. 2019 Sep 23;7:e7606. doi: 10.7717/peerj.7606 (PMC6761920; doi:10.7717/peerj.7606)
Supplement: Supplemental Information 10 — CAR681 Howland Forest project site information is provided including a map of the project area with approximate locations for CAR681 inventory plots and location of flux towers. Links to CAR681 project data for the cumulative performance report and the Project Design Document are provided. [file peerj-07-7606-s010.docx]

**Supplement S8. Howland Forest Research Forest CAR681 Project Details**

**
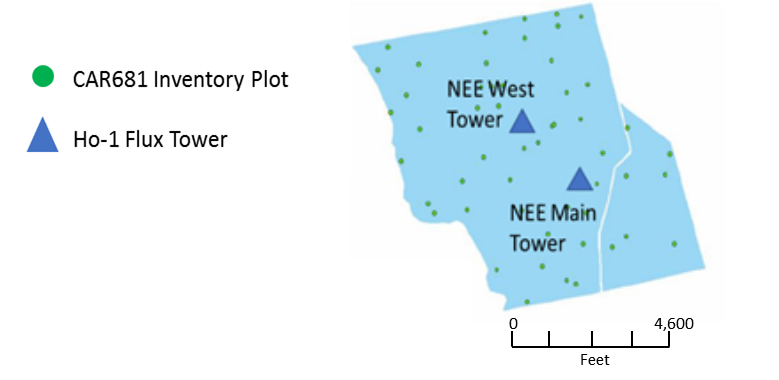
**

Fig. 5 Redrawn from: Project Design Document (12-02-2014) showing CAR681 forest inventory plot sites with flux tower approximate locations added. Source:

<https://thereserve2.apx.com/mymodule/reg/TabDocuments.asp?r=111&ad=Prpt&act=update&type=PRO&aProj=pub&tablename=doc&id1=681>

For additional flux tower information see: Hollinger et al. 2004

Project webpage link: <https://thereserve2.apx.com/mymodule/reg/TabDocuments.asp?id1=681&action=&ad=Prpt&act=update&sBtn=&Type=PRO&r=111&tablename=doc&aProj=pub>


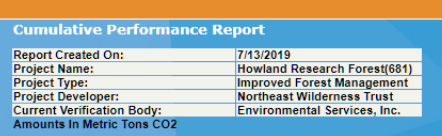


CAR681 Webpage Cumulative Performance Report: <https://thereserve2.apx.com/myModule/rpt/myrpt.asp?r=802&md=Prpt&id1=%20681>


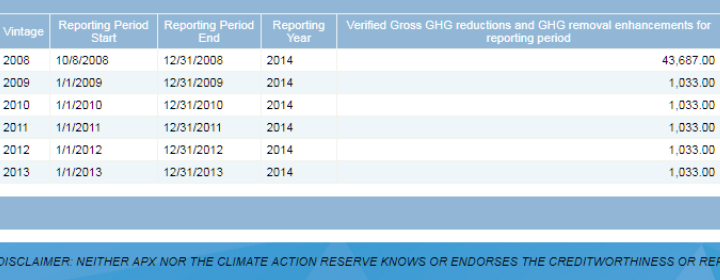


*DISCLAIMER: NEITHER APX NOR THE CLIMATE ACTION RESERVE KNOWS OR ENDORSES THE CREDITWORTHINESS OR REPUTATION OF ANY CLIMATE ACTION RESERVE ACCOUNT HOLDER LISTED IN THIS DIRECTORY*
